# Clinicopathological Concordance in 2216 Cases of Skin Biopsy over One Year: An Indian Experience

**DOI:** 10.7759/cureus.7752

**Published:** 2020-04-20

**Authors:** Akanksha Malik, Fouzia Siraj, Sharma Shruti, Pooja Gupta, Geeti Khullar, V Ramesh

**Affiliations:** 1 Pathology, All India Institute of Medical Sciences, Rishikesh, IND; 2 Pathology, National Institute of Pathology, New Delhi, IND; 3 Dermatology, Vardhman Mahavir Medical College and Safdarjung Hospital, New Delhi, IND

**Keywords:** skin biopsy, concordance, clinicopathological

## Abstract

Introduction

To determine the spectrum of diseases and the level of clinicopathological concordance in skin biopsies received over a period of one year.

Methods

A total of 2216 skin biopsy cases received over a period of one year at a tertiary care center were retrospectively analyzed. The cases were further divided into further categories in levels of concordance based on the agreement between the clinical and histopathological diagnosis rendered.

Results

Of the cases, 61.01% showed clinicopathological concordance. Cases with a descriptive pathological diagnosis, not matching the clinical diagnosis, constituted 31.54%, whereas 4.02% of cases had a definitive pathological diagnosis, which was discordant with the clinical differentials; 3.29% biopsies were inadequate.

Conclusion

This study highlights the clinicopathological concordance in all the biopsies received from dermatology. It emphasizes the importance of skin biopsies in arriving at the diagnosis. However, it is a tool that must be used judiciously. Skin biopsies are also pivotal in flagging malignancies that may mimic benign lesions.

## Introduction

Skin biopsy is an irreplaceable diagnostic tool in dermatological diseases. It also remains the gold standard investigation, however, a number of variables impact the interpretation of skin biopsies [1]. Skin biopsies are performed most often either to arrive at a conclusive diagnosis or to evaluate the response to therapy [2]. Additionally, the biopsies may be performed for medico-legal reasons to confirm clinical suspicion. The biopsies may also be performed to affirm the clinical diagnosis, especially in cases that might not respond to the chosen therapy [3].

## Materials and methods

A retrospective study was undertaken in a tertiary care center where 2216 skin biopsy cases were analyzed over a period of one year, ranging from June 2018 to May 2019. The patient demographics, site of biopsy, type of biopsy, provisional diagnosis, and pathological diagnosis rendered were compiled from the archived requisition forms and slides.

The slides were reported and reviewed by two pathologists and one dermatologist.

The provisional and pathological diagnoses were divided into tissue pattern reaction categories as described in Weedon’s skin pathology textbook [4]. The pathological diagnoses were evaluated for concordance with the clinical diagnosis and were placed in the following categories:

Category 1: Concordant definitive pathological and clinical diagnosis

Category 2: Descriptive pathological diagnosis consistent with the clinical diagnosis

Category 3: Descriptive pathological diagnosis non-consistent with the clinical diagnosis, however, the findings do not amount to any definitive diagnosis

Category 4: Definitive pathological diagnosis non-consistent with the clinical diagnosis.

Category 5: Inadequate sample

Category 6: Cases in which no clinical diagnosis was mentioned

Categories 1 and 2 were taken to be clinically concordant, whereas, Categories 3 and 4 were deemed to be discordant.

Category 6 was not evaluated for concordance since no clinical diagnosis was provided. However, these cases were included in the analysis of other parameters.

Descriptive statistics were used.

## Results

Demographics

**Figure 1:**
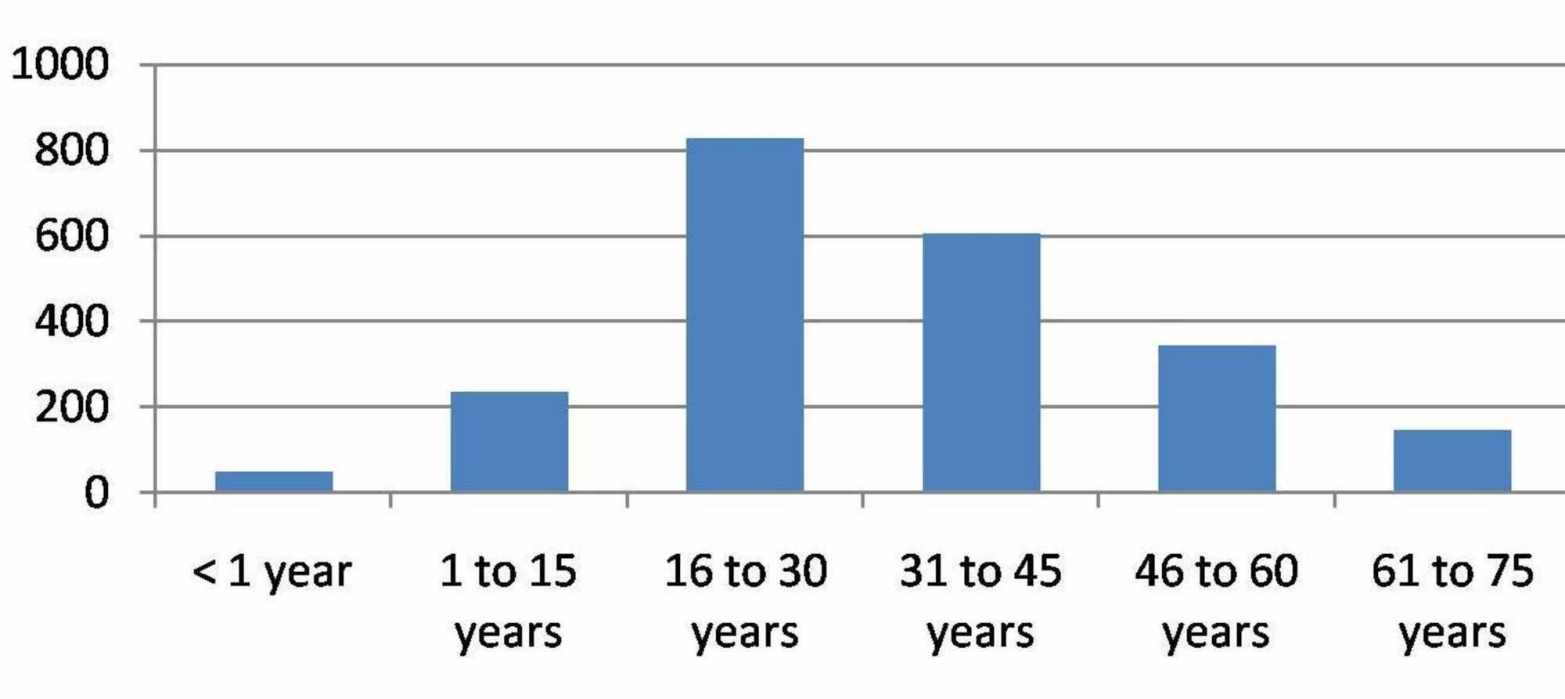
Age-wise distribution of cases

The patient age groups are given in Figure 1. The male:female ratio was 1.16:1.

The most common site of biopsies was the upper limb, whereas the perianal region was the least common. The most common provisional diagnosis suggested in the requisition form was Hansen's disease followed by lichenoid dermatoses.

One-thousand twelve (1012; 45.67%) of the cases were found to have definitive pathological clinical concordance (Category 1). Category 2 constituted 340 (15.34%) cases. We considered the summation of both these groups as concordant, that is, 61.01 %. Category 3 constituted 699 (31.54%) cases whereas 89(4.02%)cases were grouped under Category 4. Seventy-three (73; 3.29%) cases that were deemed inadequate fell into Category 5. Category 6 included 0.14% cases where no clinical diagnosis was provided, therefore, these could not be evaluated for concordance. See Table 1. However, these cases were included in the analysis of other parameters.

**Table 1:** Category-wise distribution of cases

Category	Frequency (%)
1	45.67
2	15.34
3	31.54
4	4.02
5	3.29
6	0.14
Total	100

Among Category 5 (inadequate), the most frequent site of a biopsy was the lower limb (25/73) and the most common clinical suspicion in these cases was panniculitis (15/73).

In the broad category of inflammatory dermatoses, the concordance was 56.35%, 73.53%, and 65.13%, respectively, for lichenoid dermatitis, spongiotic dermatitis, and psoriasiform dermatitis. Vesiculobullous diseases and vasculopathic disorders were in agreement with the clinical diagnosis in 55.36% and 45.9% cases, respectively. Granulomatous diseases were divided into Hansen's disease, tubercular, and non-infectious granulomas, which showed a concordance of 51.3%, 70.77%, and 40%, respectively. Type I and type II reactions of leprosy showed concordance rates of 100% and 76.66%, respectively. Pigment disorders showed a concordance rate of 87.82%. Among tumors, the benign lesions showed a clinical-pathological agreement in 68.5%, whereas malignant tumors showed 57.15% concordance. The maximum number of cases that were grouped under Category 4 belonged to benign tumors (37/89).

Among the final pathological diagnoses rendered, the most common were descriptive reports, which included non-specific features like mild spongiosis of epidermis and superficial perivascular inflammation. This was followed by granulomatous lesions.

The cases with their categories are summarized in Table 2.

**Table 2:** Distribution of clinical diagnosis into categories Erythema nodosum leprosum (ENL), Tuberculosis (TB), Granuloma annulare (GA), Post-Kala-Azar dermal leishmaniasis (PKDL)

Clinical diagnosis	Category (1+2)	Category 3	Category 4	Category 5	Total
Inflammatory dermatitis					
Lichenoid dermatitis	182 [56.35%]	126[39.01%]	7 [2.17%]	8 [2.48%]	323
Psoriasis and psoriasiform	99 [65.13%]	46 [30.26%]	3 [1.97%]	4 [2.63%]	152
Spongiotic	50 [73.53%]	16 [23.53%]	1 [1.47%]	1 [1.47%]	68
Vesicullobullous	31 [55.36%]	22 [39.29%]	1 [1.79%]	2 [3.57%]	56
Vasculopathic disorders	28 [45.9%]	30 [49.18%]	2 [3.28%]	1 [1.64%]	61
Panniculitis	20 [40%]	15 [30.00%]	0 [0.00%]	15 [30.00%]	50
Granulomatous lesions					
Infectious					
Hansen’s disease	204 [51.13%]	185[46.37%]	0 [0.00%]	10 [2.51%]	399
ENL	23 [76.66%]	7 [23.33%]	0 [0.00%]	0 [0.00%]	30
Type I reaction	11 [100%]	0 [0.00%]	0 [0.00%]	0 [0.00%]	11
TB	46 [70.77%]	17 [26.15%]	0 [0.00%]	2 [3.08%]	65
Non-infectious [GA/Sarcoidosis]	20 [40%]	22 [44.00%]	5 [10.00%]	3 [6.00%]	50
Non-granulomatous Infections [Fungal/PKDL]	27 [33.33%]	42 [51.85%]	7 [8.64%]	5 [6.17%]	81
Pigment disorders	173 [87.82%]	21 [10.66%]	1 [0.51%]	2 [1.02%]	197
Disorders of hair shaft					
Folliculitides	5 [83.3%]	1 [16.67%]	0 [0.00%]	0 [0.00%]	6
Alopecia	44 [88%]	5 [10.00%]	0 [0.00%]	1 [2.00%]	50
Scar/Keloid	10 [76.9%]	1 [7.69%]	2 [15.38%]	0 [0.00%]	13
Sclerosing disorders	58 [73.42%]	18 [22.78%]	1 [1.27%]	2 [2.53%]	79
Cysts	26 [74.82%]	4 [11.43%]	5 [14.29%]	0 [0.00%]	35
Pseudolymphoma/Lymphoid proliferation	5 [83.33%]	1 [16.67%]	0 [0.00%]	0 [0.00%]	6
Cutaneous deposits	13 [29.5%]	28 [63.33%]	1 [0.02%]	2 [0.045%]	44
Cutaneous infiltrates - non-lymphoid	6 [54.5%]	2 [18.18%]	2 [18.18%]	1 [9.09%]	11
Perforating disorders	7 [30.43%]	15 [65.22%]	1 [4.35%]	0 [0.00%]	23
Tumors					
Benign	174 [68.5%]	37 [14.5%]	37 [14.5%]	6 [0.024%]	254
Malignant	27 [57.15%]	13 [26.53%]	5 [10.20%]	3 [6.12%]	49
Premalignant	7 [50%]	5 [35.71%]	1 [7.14%]	1 [7.14%]	14
Nevus	42 [68.85%]	9 [14.75%]	7 [11.48%]	3 [4.92%]	61
No diagnosis mentioned	4 [57.15%]	0 [0.00%]	2 [28.57%]	0 [0.00%]	7
Miscellaneous	12 [57.14%]	9 [42.86%]	0 [0.00%]	0 [0.00%]	21

## Discussion

The diagnosis of skin diseases is often challenging clinically; this can be aided by the use of biopsies. The histopathological picture depends on a variety of factors that influence the diagnosis; this has been illustrated in various studies. These include the clinical information provided, age of the lesion, technique of the biopsy, and fixation and processing of the tissue amongst others [5].

Few studies have been undertaken to evaluate the utility of skin biopsies in the past, with the majority focusing on a specific disease entity. Aslan et al. found the clinicopathological agreement to be 76.8% whereas Balasubramanian et al. found the same to be 59.8% and partial concordance in their study was recorded to be 7.6% [1,6]. In a Malaysian experience of 400 biopsies, Yap et al. recorded a clinicopathological concordance of 86.8% [7]. In a study of 282 cases, Gupta et al. found the same to be 85.8% of the cases, whereas a Greek study found the same to be 68% [5,8]. The clinicopathological concordance in our study was found to be 61.01%.

The majority of the studies that have been undertaken have focused on specific pathologies. Mehta et al., in a study of 100 cases of psoriasis and psoriasiform disorders, found the clinicohistopathological correlation in 57 cases, whereas our study found that 65.13% of the cases with the provisional diagnosis of psoriasis showed features consistent with the disease [9]. Balasubramanian et al. found a similar concordance of 68.2% [1].

The concordance of lichenoid tissue reactions in our study was 56.35%, which was in contrast to many studies in the literature where the same was found to be in the range of 70.6% and 87.2% [1,10].

Category 3 (descriptive reports without any definitive diagnosis) accounted for 39% and 30.26% of lichenoid and psoriasiform groups, respectively, in our study. The discrepancy of agreement in these inflammatory diseases could be attributed to the biopsy being taken from a non-representative lesion or an early or late stage of the disease.

In our study, the clinical and histological diagnosis in Hansen's disease showed a concordance of 51.3%, as compared to 58.8% in the study by Balasubramanian [1]. In our cases, the subtype of leprosy with the maximum concordance was found to be borderline tuberculoid (BT) Hansen's followed by lepromatous leprosy (LL) Hansen's. Similar findings were published by Manandhar et al. who found the greatest concordance in BT Hansen's followed by LL Hansen's [11]. However, Bhatia et al. and Balasubramanian et al. found the maximum concordance in LL Hansen's followed by BT Hansen's [1,12]. Our study had relatively more number of cases with the provisional diagnosis of BT Hansen's as compared to other subtypes, therefore, it may have biased the finding. Out of 65 cases with one of the clinical differential diagnoses being cutaneous tuberculosis, 70.77% cases showed clinicopathological concordance. Dutta et al., in a study of granulomatous lesions, found the same to be 91.6% in 24 cases of cutaneous tuberculosis [13].

The clinical suspicion of malignancy was raised in 49 cases out of which 28 were confirmed on histopathology (57.15%). Another Indian study found the agreement to be 52.7% as compared to 89.2% in Western literature [1,6]. In this group, the most commonly encountered malignancy was basal cell carcinoma (BCC), which showed a concordance of 79.16%. A similar percentage was noted in previous studies for BCC concordance ranging from 92.6% to 85.4% [6,14]. We received only one case of melanoma that had the histopathological features of the same. However, we received a very small number of biopsies with the clinical diagnosis of melanoma, as it is uncommon in our population, therefore, we cannot comment on the concordance of this entity. We also received a very small number of cases of squamous cell carcinoma (SCC), as these are usually referred from surgical oncology in our hospital. The majority of the cases that came with the differential of SCC showed features of carcinoma in situ, perhaps owing to the small size of biopsy in a large lesion.

Benign tumors showed clinicopathological agreement in 68.5% of the cases. Cysts were concordant in 74.82%. Sellheyer et al. found the concordance of benign and cystic lesions to be 80% [14].

Sixty-eight point five (68.85%) of melanocytic nevi showed clinicopathological agreement; this was less than the study by Aslan et al., which showed the same to be 88% [6].

Seborrheic keratosis was most commonly misdiagnosed clinically as benign nevi in 4/61 cases. Parslew et al. also reported seborrheic keratosis to be a frequent misdiagnosis in clinically suspected cases of benign nevi, which is an established clinical problem [15]. In literature, the clinicopathological consistency of seborrheic keratosis has been recorded as 71% and 88% [1,6]. In our study, seborrheic keratosis showed a concordance of 90%.

In our study, Category 4 (definitive pathological diagnosis inconsistent with clinical diagnosis) constituted 89/2216 (4.02%) cases. The cases included in this category were either misdiagnosed clinically or had differing therapeutic implications. Benign tumors comprised 41.5% (37/89) in this category. Three of these cases were finally diagnosed as molluscum contagiosum, which had come with the clinical diagnoses of sebaceoma, poroma, and hidrocystoma, respectively. Four cases clinically diagnosed as benign tumors showed a premalignant lesion or malignancy on histopathology viz. two cases of basal cell carcinoma (BCC), one atypical melanocytic lesion, and one carcinoma in situ, which were clinically diagnosed as acquired nevus, angiokeratoma, dermatofibroma, and pyogenic granulomas, respectively. This constituted 0.015% of the benign lesions. Parslew et al., in their study of 1000 benign tumors, found malignancies clinically misdiagnosed as benign tumors to be 0.9% [15].

The lack of a wider differential diagnosis panel in clinically ambiguous cases may have contributed towards an increased number of cases being grouped under Category 3. In the authors’ personal experience, there was ease in reaching a diagnosis where photographs of the lesion were taken by the referring dermatologist so that better correlation could be done at the time of reporting the cases. In a study by Wong et al. in 2015, 57% of the respondent dermatopathologists emphasized the lack of clinical information and differential diagnoses as a potential pitfall for adequate evaluation of skin biopsies. They also stressed the utility of an array of differential diagnosis over clinical descriptions when the biopsies were sent by dermatologists, however, the opposite was preferred if the biopsy was requested by a general practitioner [16].

## Conclusions

Our study is one of the few studies that highlight the clinicopathological concordance in all the biopsies received from dermatology. It emphasizes the importance of skin biopsies in arriving at the diagnosis, however, it is a tool that must be used judiciously. Skin biopsies are also pivotal in flagging malignancies that may mimic benign lesions.

## References

[1] Balasubramanian P, Chandrashekar L, Thappa DM, Jaisankar TJ, Malathi M, Ganesh RN, Singh N (2015). A retrospective audit of skin biopsies done in a tertiary care center in India. Int J Dermatol.

[2] Farmer ER (2000). Why a skin biopsy?. Arch Dermatol.

[3] Werner B (2009). Skin biopsy and its histopathologic analysis: Why? What for? How? Part I. An Bras Dermatol.

[4] Johnston R (2016). Weedon's Skin Pathology Essentials E-Book. https://www.elsevier.com/books/weedons-skin-pathology-essentials/johnston/978-0-7020-6928-4.

[5] Korfitis C, Gregoriou S, Antoniou C, Katsambas AD, Rigopoulos D (2014). Skin biopsy in the context of dermatological diagnosis: a retrospective cohort study. Dermatol Res Pract.

[6] Aslan C, Göktay F, Mansur AT, Aydıngöz İE, Güneş P, Ekmekçi TR (2012). Clinicopathological consistency in skin disorders: a retrospective study of 3949 pathological reports. J Am Acad Dermatol.

[7] Yap B, Boon F (2009). Dermatopathology of 400 skin biopsies from Sarawak. Indian J Dermatol Venereol Leprol.

[8] Gupta P, Karuna V, Grover K, Rathi M, Verma N (2018). The histopathological spectrum of skin diseases with emphasis on clinicopathological correlation: a prospective study. J Diagn Pathol Oncol.

[9] Mehta S, Singal A, Singh N, Bhattacharya SN (2009). A study of clinicohistopathological correlation in patients of psoriasis and psoriasiform dermatitis. Indian J Dermatol Venereol Leprol.

[10] Hegde VK, Khadilkar UN (2014). A clinicopathological study of interface dermatitis. Indian J Pathol Microbiol.

[11] Manandhar U, Adhikari RC, Sayami G (2013). Clinico-histopathological correlation of skin biopsies in leprosy. Journal of Pathology of Nepal.

[12] Bhatia AS, Katoch K, Narayanan RB, Ramu G, Mukherjee A, Lavania RK (1993). Clinical and histopathological correlation in the classification of leprosy. Int J Lepr Other Mycobact Dis.

[13] Dutta B, Baruah RR, Huda MM, Gogoi BC, Dutta A (2016). A clinicopathological study of cutaneous granuloma. J Evolution Med Dent Sci.

[14] Sellheyer K, Bergfeld WF (2005). A retrospective biopsy study of the clinical diagnostic accuracy of common skin diseases by different specialties compared with dermatology. J Am Acad Dermatol.

[15] Parslew RA, Rhodes LE (1997). Accuracy of diagnosis of benign skin lesions in hospital practice: a comparison of clinical and histological findings. J Eur Acad Dermatol Venereol.

[16] Wong C, Peters M, Tilburt J, Comfere N (2015). Dermatopathologists’ opinions about the quality of clinical information in the skin biopsy requisition form and the skin biopsy care process: a semiqualitative assessment. Am J Clin Pathol.

